# Psychological Support for Personality (PSP) versus treatment as usual: study protocol for a feasibility randomized controlled trial of a low intensity intervention for people with personality disorder

**DOI:** 10.1186/s13063-018-2920-0

**Published:** 2018-10-10

**Authors:** Mike J Crawford, Lavanya Thana, Jennie Parker, Oliver Turner, Kwek Pei Xing, Mary McMurran, Paul Moran, Timothy Weaver, Barbara Barrett, Amy Claringbold, Paul Bassett, Rahil Sanatinia

**Affiliations:** 1 Personality Disorder Research Unit, Centre for Psychiatry, Imperial College London, London, UK; 2 Department of Medicine, Division of Brain Sciences, Centre for Psychiatry, 7th Floor Commonwealth Building, Hammersmith Hospital Campus, Du Cane Road, London, W12 0NN UK; 3 Research and Development Department, Central and North West London NHS Foundation Trust, Stephenson House, 75 Hampstead Road, London, NW1 2PL UK; 4 Barnet, Enfield and Haringey NHS Foundation Trust, St Ann’s Hospital, St Ann’s Road, Haringey, London, N15 3TH UK; 5 Section of Forensic Mental Health, University of Nottingham, Nottingham, NG7 2UH UK; 6 School of Social and Community Medicine, Bristol University, Oakfield House, Oakfield Grove, Bristol, BS8 2BN UK; 7 Mental Health Social Work & Integrative Medicine, Middlesex University, The Burroughs, Hendon, London, NW4 4BT UK; 8 Centre for the Economics of Mental and Physical Health, King’s College London, David Goldberg Centre, De Crespigny Park, London, SE5 8AF UK; 9 Statsconsultancy Limited, 40 Longwood Lane, Amersham, Buckinghamshire HP7 9EN UK

**Keywords:** Personality disorder, Psychological treatment, Low intensity, Brief intervention, Suicidal behavior, Randomized trial

## Abstract

**Background:**

Previous research has demonstrated the clinical effectiveness of long-term psychological treatment for people with some types of personality disorder. However, the high intensity and cost of these interventions limit their availability. Lower-intensity interventions are increasingly being offered to people with personality disorder, but their clinical and cost effectiveness have not been properly tested in experimental studies. We therefore set out to develop a low intensity intervention for people with personality disorder and to test the feasibility of conducting a randomized controlled trial to compare the clinical effectiveness of this intervention with that of treatment as usual (TAU).

**Methods:**

A two-arm, parallel-group, single-blind, randomized controlled trial of Psychological Support for Personality (PSP) versus TAU for people aged over 18 years, who are using secondary care mental health services and have personality disorder. We will exclude people with co-existing organic or psychotic mental disorders (dementia, bipolar affective disorder, delusional disorder, schizophrenia, schizoaffective disorder, or schizotypal disorder), those with cognitive or language difficulties that would preclude them from providing informed consent or compromise participation in study procedures, and those who are already receiving psychological treatment for personality disorder. Participants will be randomized via a remote system in a ratio of PSP to TAU of 1:1. Randomization will be stratified according to the referring team and gender of the participant.

A single follow-up assessment will be conducted by masked researchers 24 weeks after randomization to assess mental health (using the Warwick and Edinburgh Well-Being Schedule), social functioning (using the Work and Social Adjustment Scale), health-related quality of life (EQ-5D-5 L), incidence of suicidal behavior, satisfaction with care (Client Satisfaction Questionnaire), and resource use and costs using a modified version of the Adult Service Use Schedule. In addition to this, each participant will be asked to complete the patient version of the Clinical Global Impression Scale.

Feasibility and acceptability will primarily be judged by study recruitment rate and engagement and retention in treatment. The analysis will focus principally on descriptive data on the rate of recruitment, characteristics of participants, attrition, adherence to therapy, and follow-up. We will explore the distribution of study outcomes to investigate assumptions of normality in order to plan the analysis and sample size of a future definitive trial.

**Discussion:**

Most people with personality disorder do not currently receive evidence-based interventions. While a number of high intensity psychological treatments have been shown to be effective, there is an urgent need to develop effective low intensity approaches to help people unable to use existing treatments. PSP is a low intensity intervention for individuals, which was developed following extensive consultation with users and providers of services for people with personality disorder. This study aims to examine the feasibility of a randomized trial of PSP compared to TAU for people with personality disorder.

**Trial registration:**

ISRCTN Registry, ISRCTN14994755. Registered on 18 July 2017.

## Background

Personality disorders are long-term mental health conditions which affect 4–7% of the general population [1]. People with personality disorder have severe problems in their relationships with others which can lead to poor mental health, social exclusion, and impaired quality of life [2, 3]. No drugs are currently licensed for the treatment of personality disorder; instead clinical guidelines recommend structured psychological therapies [4, 5]. Evidence-based psychological treatments for people with personality disorder are intensive. They typically combine individual and group-based therapy delivered over a 12–18-month period [6, 7]. Most people with personality disorder do not have access to these intensive interventions and even when they are available as many as half of those who are referred do not engage with them [8, 9]. As a result most people with personality disorder do not receive evidence-based treatment [10]. Usual care for people with personality disorder is often inconsistent and many service users report negative experiences of the care they receive [11–13].

Current guidelines for treating depression and other common mental disorders generally recommend a “stepped care approach” in which all patients are initially offered a low-intensity intervention and only those who do not respond are offered longer and more intensive treatments [14–16]. Low intensity intervention is not a substitute for more intensive treatments but rather an approach which aims to increase access to appropriate care and improve equity of access to more costly and intensive interventions [15].

It has been argued that a stepped care approach should also be used to treat people with personality disorder [17]; however, little research has been undertaken to develop or test “low intensity” interventions for people with personality disorder. As a result, very few people with personality disorder receive evidence-based interventions.

A systematic search for clinical trials of psychosocial interventions for people with borderline personality disorder published in 2014, found evidence from two small-scale studies that had examined the impact of short-term advice and support [7]. Both studies were conducted in the United States. The first examined the impact of a single session of psychoeducation about personality disorder and its treatment [18] and the second involved six sessions of manual-assisted cognitive therapy compared to treatment as usual (TAU) [19]. Both studies reported short-term benefits associated with these low intensity interventions. Since the publication of this review, a further study has examined the impact of providing people with borderline personality disorder access to a web-based psychoeducational resource which included information about symptoms of the condition, its etiology, and prognosis [20]. A total of 80 women with borderline personality disorder were recruited from the Internet. Those given access to the resource showed greater improvements in mental health, measured using the Zanarini Rating Scale for Borderline Personality Disorder, compared to those who were not. [20]. In contrast to the promising results of these studies, no benefit was seen among people with personality disorder who were offered a short-term group-based problem-solving therapy for people with personality disorder [21].

Concerns have been raised about the potential for negative effects associated with the use of short-term interventions for people with personality disorder [4]. A secondary analysis of data from a brief manualized form of cognitive behavior therapy for people with repeated deliberate self-harm found that costs of care among people with personality disorders were higher among those offered the brief intervention compared to those offered standard care [22]. Current clinical guidelines for the treatment of people with borderline personality disorder in the UK specifically caution against the use of interventions lasting less than three months [4].

Despite these recommendations, mental health services are under increasing pressure to offer low intensity interventions to people with personality disorder. Such interventions consist of a limited number of sessions of psychoeducation and structured psychological support. In keeping with recommendations of experts, particular attention is paid to the way interventions are delivered, including making sure that staff delivering them are properly supervised [23]. Such interventions aim to help patients make links between their emotions and actions and to improve people’s ability to care for themselves [24, 25]. While this approach has the potential to increase access to care and improve patient outcomes, its clinical and cost effectiveness is unknown.

The purpose of this study is to examine the feasibility of using a randomized trial to test the clinical and cost effectiveness of a low intensity intervention for people with personality disorder.

### Research objectives

The main objective of the study is to find out whether it is feasible to conduct a randomized controlled trial of Psychological Support for Personality (PSP) versus TAU for people in contact with mental health services who have personality disorder. The secondary objectives of the study are:

1. To conduct a parallel-arm, single-blind, randomized trial of a psychologically informed low intensity intervention for people with personality disorder;
2. To determine the uptake and delivery of both the low intensity intervention and TAU, including the number of sessions offered and attended;
3. To determine the rate of recruitment, distribution of outcomes, rate of follow-up, and extent of clustering between practitioners to inform the sample size calculation for a future definitive trial;
4. To explore service user and provider beliefs about the acceptability and design of the trial and factors that facilitate or hinder the delivery of a low intensity intervention for people with personality disorder.

If the study demonstrates feasibility, we will use the results to seek funding for a phase III randomized trial. The results of a fully powered trial would ensure that future guidelines on the use of low intensity interventions for people with personality disorder would be based on high-quality evidence, rather than having to rely solely on the expert opinion that forms the basis of current treatment guidelines.

## Methods/Design

The PSP trial is a two-arm, parallel group, single-blind, randomized controlled trial with a 24-week follow-up assessment. The trial includes an integrated process evaluation which will explore service user and provider beliefs about the impact of this intervention, mechanisms of action, and factors that facilitate or hinder its successful delivery.

### Study setting

Study participants will be recruited from secondary care mental health services in London. Recruitment will be from community mental health teams, home treatment teams, and other community-based mental health services.

### Eligibility criteria

The target population is adults aged 18 years or over who have a clinical diagnosis of personality disorder and are able and willing to provide written informed consent to take part in the study.

The exclusion criteria are:

- A current co-existing diagnosis of an organic or psychotic mental disorder (dementia, bipolar affective disorder [type I and II], delusional disorder, schizophrenia, schizoaffective disorder, or schizotypal disorder);
- Cognitive or language difficulties that preclude individuals providing informed consent or compromise participation in study procedures;
- Current receipt of psychological treatment for personality disorder.

### Interventions

Those in the active arm of the trial will be offered PSP while those in the control arm of the trial will continue to receive TAU.

#### Psychological Support for Personality

The development of the form and content of this intervention was based on the recommendations of an expert panel of service users and providers. We used a nominal group technique to seek a consensus on the optimal form and content of the intervention [26]. Seven clinicians and six service users took part in the group. Service providers were all experienced in treating people with personality disorder and were selected to ensure a range of different professional backgrounds and training. Service user members were chosen because they had lived experience of using services for people with personality disorder. Each panel member was asked to complete a survey comprising 21 questions on the optimal form and content of a low intensity intervention for people with personality disorder. At a half-day panel meeting, all members of the group were then given an opportunity to discuss their preferences for the intervention. Following these discussions, each panel member was asked to re-rate their preferences in light of other panel members’ responses to the first round of the survey and their discussions with other panel members at the meeting. Key findings from the second-round survey are presented in Table 1. Qualitative comments from group members highlighted the importance of ensuring that the intervention was tailored to the specific needs of the participants. Given the short-term nature of the intervention, service users and providers stated that it was important to set realistic aims for the intervention and help prepare people for the end of the sessions.

**Table 1:** Views of expert panel and their impact on the design of the active intervention

Topic	Views of panel members	Feature of the active intervention
Name of the intervention	• Avoid using the term 'brief' which may give the impression the person does not have serious problems. • The word 'support' was preferred to the words 'intervention' or ‘treatment’ as this recognises that people are receiving help to better self-manage rather than something that happens to them.	Decision to call the intervention ‘Psychological Support for Personality’.
Target group	• To try to keep this as broad as possible, because many people with personality disorder are excluded from services due to coexisting conditions.	To make the intervention available to people with coexisting, non-psychotic, axis I disorders including alcohol and drug misuse.
Number of sessions	• Should generally offer six to 10 sessions. • The panel recommended that no minimum or maximum number of sessions be set.	That the therapist and service user agree the number of sessions to be delivered following the initial assessment - but (in general) to offer six to 10 sessions.
Contact outside of sessions	• The panel recommended that limited telephone contact in times of crisis could be beneficial as general crisis support lines were often experienced as unhelpful. • Service user members expressed the view that telephone-based sessions were not likely to be as helpful as face-to-face meetings, but felt that telephone-based sessions should be offered as an alternative to face meetings if this was the service user’s preference.	To offer limited access to telephone support at times of crisis. Sessions to generally be offered face-to-face with the option of telephone based sessions if service users prefer this.
Missed sessions	• Service users felt that, if advance notice was given, people should be allowed to re-schedule sessions; clinicians agreed with this but felt that a limit needed to be placed on the number of sessions that could be cancelled.	The therapist should reschedule session(s) if asked to do so by the service user. Missed sessions may be substituted, therapists should use discretion when deciding this.
Provision of group-based sessions	• Concerns were expressed about the impact of isolated peer group sessions in the context of a short term individual intervention. • There was a consensus that it would be appropriate to refer people to community-based groups and other resources if these are available.	Not to incorporate group sessions within the intervention, but to refer service users to community-based groups outside of the intervention if these are available.
Use of diagnostic term ‘personality disorder’	• Two thirds of the group were in favour of the use of the term and a third were not, unless discussion was initiated by the service user. • All agreed that providing information about personality and personality-related problems was an important part of the intervention. • The group recommended the use of a shared formulation to give people a framework for trying to understand their problems and what might help them.	The intervention includes a discussion of personality and the origins of personality-related problems. The intervention includes the development of a shared formulation. Personality disorder diagnosis is discussed but only used if the person finds this helpful.
Liaison with primary care	• Service user members stated that the extent to which details of the intervention were shared with their GP should depend on the quality of their relationship with them. • Most staff members of the group stated that information about the intervention should be shared with the GP.	Copies of the shared formulation and final discharge plan will be shared with the service users GP if they agree to this.
Medication	• Clinicians stated that it would complicate the intervention to include a routine review of medication. • Service users expressed a preference for including a review either before the intervention or at an early stage. • All participants were concerned about the impact of side effects of medication on peoples’ health.	To offer a review of medication to those who are concerned about the psychotropic medication they are taking.
Involving significant others	• Panel members agreed that this could be helpful but emphasised the importance of ensuring that this was what the service user wanted and making sure that service users did not feel left out of any joint sessions.	To offer a joint meeting with a significant other if the service user would like this.
Treatment goals	• All members supported the development of person-centred aims for the intervention. • Service users members stated that the word 'goals' was not ideal and may inadvertently lead people to focus on what they had *not* achieved during the intervention. • There was general agreement about the need to manage expectations and having flexibility in reviewing progress during the intervention.	Therapists to ensure that service users are provided with information about the limited focus of the intervention. Service users and therapists to agree a shared focus (generally by the end of the second session).
Psychological approaches	• Service users members emphasised the importance of psychoeducation to help people understand and cope with stigma and self-stigma. • Members of the panel stated that other approaches such as mindfulness and problem-solving could be beneficial depending on the person’s individual needs.	Psychoeducation to be the starting point for the intervention.

The results of the nominal group meeting were presented to managers of the service which would deliver the treatment and further feedback used to develop treatment guidelines for the intervention. Key features of PSP are presented in Table 2, together with comparative information on high intensity psychological treatments for people with personality disorder. Further details are available on request from the corresponding author. PSP consists of 6–10 sessions of psychoeducation and psychologically informed support for individuals. The exact number of sessions, frequency, and duration offered will be based on clinical judgment and patient preferences, but we anticipate that up to ten sessions will be offered over a 3–6-month period and that each session will last for 30–45 min.

**Table 2:** Summary of key features of Psychological Support for Personality (PSP) compared to other psychological interventions tested in clinical trials

Feature of the intervention	Psychological Support for Personality	Web-based psychoeducation [20]	Manual assisted cognitive therapy [19]	Mentalization Based Treatment [30, 31]	Dialectical Behaviour Therapy [28, 29]
Target group	Adults with personality disorder	Women with borderline personality disorder	Adults with borderline personality disorder who self-harm	Adults with borderline personality disorder	Adults with borderline personality disorder
Mode(s) of delivery	Individual sessions plus telephone support	Access to web-based psychoeducation	Individual sessions plus booklet	Group and individual sessions plus telephone support	Group and individual sessions plus telephone support
Content of sessions	Information on personality, personality disorder, validation and acceptance. Tailored psychological support aimed at promoting metalizing and distress tolerance	Information about borderline personality, disorder and role of psychological and pharmacological treatments	Strategies for emotional regulation, problem-solving, management of negative thinking and substance misuse	Methods for promoting mentalizing and improving a person’s mental health and interpersonal functioning	Behavioural skills coaching in areas including mindfulness, emotion regulation and distress tolerance
Delivered by	Clinical staff who are experienced in working with people with personality disorder and receive regular supervision	Content of website developed by clinicians with expertise in borderline personality disorder	Clinical staff who have received a three-day and receive regular supervision	Clinical staff who have received a minimum of three days training and receive regular supervision	Mental health professionals who have been trained to deliver DBT and receive regular supervision
Frequency of sessions	Flexible (usually once a week or one a fortnight)	Service users able to access website whenever they choose	Weekly sessions	Weekly individual sessions and weekly groups	Weekly individual sessions and weekly groups. Telephone consultations as required.
Length of sessions	45 to 60 minutes	Determined by the service user	30 to 60 minutes	Groups last 75 to 90 minutes	60 minute individual sessions 150 minute groups
Length of treatment	Flexible (three to six months)	Determined by the service user	Six weeks	18 months	12 to 18 months
Total number of sessions	Six to ten sessions	Determined by the service user	Six sessions	100 to 150 sessions*	100 sessions*

*Exact number and length of sessions varies in clinical practice. These data are from publications of randomised trials of the interventions

The intervention will be delivered in accordance with recommendations for psychosocial interventions for people with personality disorders [25] and low intensity interventions for people with other non-psychotic mental disorders [27].

During the first two sessions, the therapist will assess the patient’s mental health, personality difficulties, and existing understanding of their problems and coping strategies in order to formulate a treatment plan, including a crisis plan. By the end of session two, the participant and the therapist will agree the focus for the remaining sessions and share this in writing with their general practitioner (GP).

The focus of the remaining sessions will depend on the needs and preferences of the participant but may include help with developing coping skills, support to better understand problems in relationships, or encouragement and advice around the person’s social and occupational needs. During these sessions, the therapist will discuss the nature of personality disorders, what leads people to develop disturbed interpersonal functioning, and what steps people can take to lessen the impact that aspects of their personality can have on their quality of life. Psychological support will draw on the two longer-term evidence-based treatments that are most widely used in the UK: Dialectical Behavior Therapy [28, 29] and Metallization-Based Treatment [30, 31]. During sessions, therapists will seek to “validate” the patient’s experience. Validation involves the therapist acknowledging that a person’s feelings, thoughts, and behaviors are valid and understandable [28]. Therapists will use a metalizing stance which involves acknowledging that the mental states of others can only be understood through curious enquiry and seeks to encourage people to play an active role in understanding how mental states can affect their thoughts, feelings, and actions and those of others [30]. Attention will also be paid to what healthcare services can and cannot do to assist people with personality disorders and support the patient in developing steps that they can take to look after their own mental health. Treatment delivery mode will be flexible, depending on patient preference, and will include individual face-to-face and telephone contact and may be supplemented by texts, emails, and liaison in other services. Patients will be provided access to written and/or web-based information and signposted to other services as appropriate.

Following delivery of PSP, the aim will be to discharge the study participant from secondary care mental health services. This aim will be made explicit to potential participants before they enrol in the study. Therapists delivering PSP will be able to refer participants to longer-term psychological treatments or continuing care from mental health services if appropriate to do so.

#### Delivering PSP

All staff delivering PSP will be registered mental health professionals with some experience of working with people with personality disorders. To ensure that therapists are appropriately supported and that the needs of the participant are being met, they will meet fortnightly as part of a multidisciplinary team to discuss progress. Staff delivering PSP will be asked to complete a short proforma for every participant that they treat which records the number and length of face-to-face, telephone, and email/text contacts they have had with patients and lists the components of the intervention they delivered during the course of the treatment. Staff will also record any participants that have withdrawn early from the PSP and the reason(s) for this (if known).

#### Treatment as usual

TAU will be delivered by staff working in community mental health teams. This comprises assessment, care planning, and review. It may involve pharmacotherapy and referral to other services including access to inpatient care at times of crisis. As part of the local care pathway for people with personality disorder, staff are asked to discuss the care pathway (including discharge from secondary care) from the outset. Staff delivering TAU will be able to refer participants to longer-term psychological treatments but any patient who is already receiving psychological treatment for personality disorders at the time of the baseline assessment will not be eligible to take part in the study.

All study participants allocated to receive TAU will complete all the study assessments. Clinical teams will not be asked to complete a proforma on any usual treatment that is being delivered; instead this information will be captured in the Modified Adult Service User Schedule at baseline and six months.

### Assessments

**Fig. 1:**
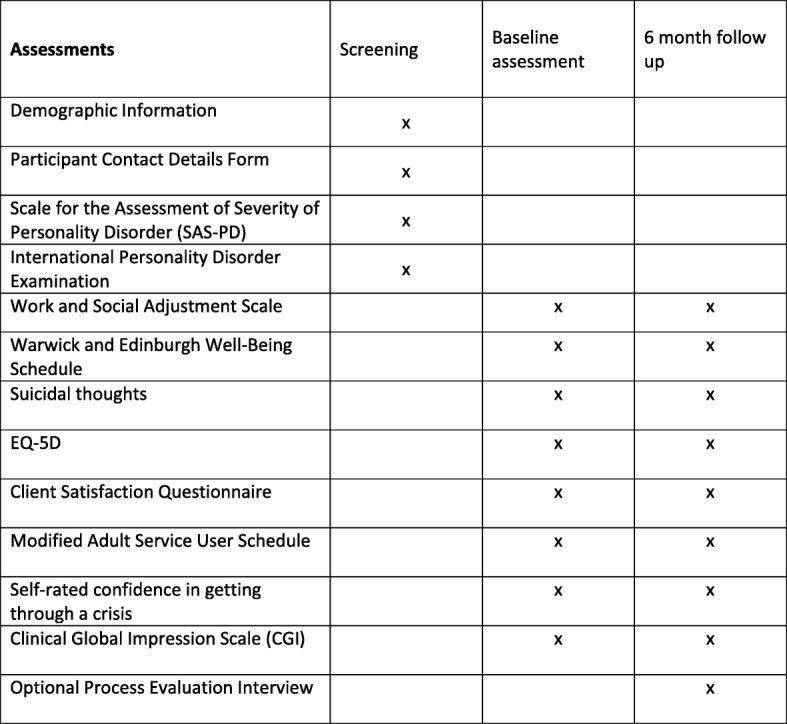
Study assessment schedule

The timing and sequence of all assessments are summarized in Fig. 1.

#### Baseline: eligibility and covariates

At baseline, we will collect demographic details and assess eligibility using the Standardized Assessment of Severity of Personality Disorder (SASPD) [32]. A score ≥ 8 on the SASPD provides a reliable assessment of the likelihood of personality disorder according to ICD-11 criteria [32]. Those who score < 8 on the SASPD will not be eligible to take part in the study. Those who are ineligible will be thanked for their time and informed of the reason(s) for this. We will also ask participants to complete the International Personality Disorder Examination (IPDE) Screening Questionnaire, a 77-item self-complete questionnaire, which provides a reliable indication of specific personality disorders using DSM-IV criteria [33]. We will use data from the IPDE to describe the range of personality problems experienced by people who take part in the study.

#### Baseline: study outcomes

Study participants will be asked to complete the following outcome measures: (1) The Work and Social Adjustment Scale (a short validated assessment of social functioning) [34]; (2) The Warwick and Edinburgh Well-Being Schedule – a seven-item questionnaire which provides a reliable assessment of mental wellbeing [35, 36]; (3) Suicidal thoughts-National Household Survey of Psychiatric Morbidity [37]; (4) the five-item EQ-5D-5 L, a reliable self-completed assessment of health-related quality of life [38] which has been shown to be sensitive to change among people with personality disorders [39]; (5) satisfaction with care will be examined using the four-item Client Satisfaction Questionnaire [40]; (6) resource use and costs will be assessed using a modified version of the Adult Service Use Schedule [41]; and (7) participants will be asked to rate any change in their mental health during the previous six months using the Clinical Global Impression Scale (CGI), a seven-point Likert scale from very much improved to very much worse [42]. Finally, participants will be asked to state how confident they are in their ability to “get yourself through difficult times and situations” on a five-point Likert scale (ranging from totally confident to totally unconfident). The psychometric properties of this item have not been tested. It was included in the study following feedback with stakeholders with lived experience of personality disorders.

#### Follow-up assessments

All study participants will complete a six-month follow-up assessment. At the six-month follow-up, the researcher will ensure that all the study outcomes that were assessed at baseline are reassessed, i.e. the Work and Social Adjustment Scale, the Warwick and Edinburgh Well-Being Schedules, suicidal thoughts and behavior using items from the National Household Survey of Psychiatric Morbidity, the EQ-5D-5 L, the Client Satisfaction Questionnaire, the Adult Service Use Schedule, patient-rated Clinical Global Impression and the five-point Likert scale on how confident they are in their ability to manage crises.

In addition to this, we will seek additional consent from study participants to take part in a qualitative interview. Following completion of the six-month follow-up interview, up to 20 participants will be invited to take part in a separate interview with a researcher with lived experience of using mental health services. During this interview, participants will be asked about their experience of taking part in the study and any steps they think we could take to improve the design of a future definitive trial. The interview will be semi-structured and guided by a topic list which will be applied flexibly. The sample for this qualitative component of the study will be purposively selected to ensure that men and women of different ages and ethnicities are selected from those in both the active and control arm of the trial. Participants from the active arm of the trial will also be selected to ensure that both those who engage and drop out of PSP are included.

### Sample size

In keeping with recommendations for feasibility studies, we have not based plans for sample size on a power calculation [43]. Instead we judged that a sample of 60 participants will generate sufficient data to assess the rate of recruitment and follow-up in a single center and estimate levels of uptake and retention in therapy among approximately 30 people in the active arm of the trial.

### Assignment of interventions

After consenting to participation and completing screening assessments, people who are found to be eligible will be randomly allocated to PSP or TAU by a clinical trial manager at the Centre for Psychiatry, Imperial College London. A randomization list will be generated using the independent web-based service “sealed envelope” (https://www.sealedenvelope.com/simple-randomiser/v1/lists). Equal numbers of participants will be randomized to the two arms of the trial and stratification will occur by gender and study center. Throughout the study, the randomization list will be encrypted and held with the Trial Coordinating Office in order to keep the study researchers blind against treatment allocation. At the end of the study the randomization list will be unencrypted and placed in the Trial Master File. Equal numbers of participants will be randomized to each arm of the trial. Randomization will be stratified according to the referring team and gender of the participant.

### Blinding

The randomization service will generate a unique trial identification number for that participant, which will be used on Case Report Forms. Research assistants will be blind to allocation status of participants, but the participants and those providing clinical care will not. Researchers will be asked to keep a contemporaneous record of any instances in which treatment allocation is disclosed to them. When a researcher is unblinded, we will arrange for a second researcher who is blinded to complete the six-month follow-up assessment.

### Study logistics

#### Recruitment

Potential participants will initially be approached about the study by any healthcare professional who is involved in their care. If staff have a patient under their care who they believe meets the eligibility criteria for the study, they will introduce the patient to the study at an appropriate time by briefly describing it to them. The patient must provide verbal agreement to discuss their eligibility and possible enrolment into the trial with a member of the research team before any further study process can take place. If a patient gives verbal agreement, a researcher will phone them to explain the study in greater detail. The researcher will then post or email a copy of the patient information sheet. Potential participants will be given no less than 24 h from receiving the information sheet to consider the information and the opportunity to question the researcher or other independent parties about their participation in the trial. Researchers will then arrange to meet potential participants to obtain written informed consent. A copy of the signed Informed Consent form(s) will be given to the patient. The original signed form(s) will be retained at the trial site. We will aim to recruit the study sample of an eight-month period.

#### Screening, baseline, and follow-up interviews

**Fig. 2:**
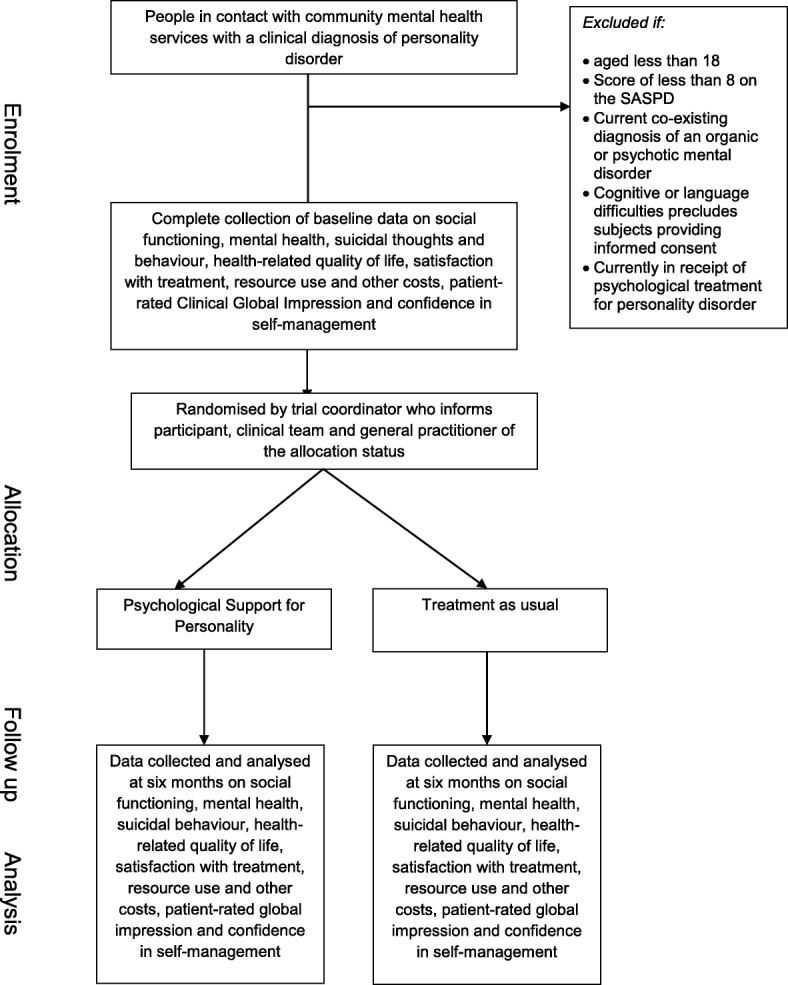
Study *flow chart*

If consent is given and documented, a Screening Assessment Case Report Form will be completed with the participant. If the participant fulfils the eligibility criteria, they will then complete the Baseline Assessment and be randomized (see Fig. 2). Following randomization, the participant’s GP and referring clinician will be informed of their enrolment into the trial.

Study participants will be contacted at three months by a study researcher to confirm contact details and as a reminder about the six-month assessment. A follow-up interview will then be scheduled for six months after randomization. All participants will be offered a £20 honorarium following completion of the six-month follow-up interview.

#### Adverse events

All adverse events (AEs) will be recorded from the time a participant gives consent until 30 days after participation. In keeping with EU guidance on the collection and verification of AE reporting in clinical trials, serious adverse events (SAE; i.e. those that result in death, are life-threatening, require hospitalization or prolongation of existing inpatients’ hospitalization, or result in persistent or significant disability or incapacity) and non-SAEs will be recorded on event forms, detailing as much of the event as possible to be reviewed and signed by Principal Investigator [44]. The Principal Investigator will assess: (1) the seriousness of the event; (2) the likelihood of causality to the event; and (3) the severity of the event. The Researcher will ask participants during any contact or scheduled visit about AEs.

The study may be subject to inspection and audit by Central and North West London NHS Foundation Trust under their remit as sponsor and other regulatory bodies to ensure adherence to GCP and the NHS Research Governance Framework for Health and Social Care.

### Data management and analysis

Data will be entered into a password-protected Microsoft Excel spreadsheet/database held on a secure server. Study data will be archived securely and then safely destroyed after 15 years. Analysis and reporting of the trial will be in accordance with CONSORT guidelines.

Data analysis will focus principally on descriptive data on recruitment rates, characteristics of participants, attrition from the trial, non-adherence to therapy, and follow-up. The effect of treatment on outcomes will be estimated on an intention-to-treat basis using differences in scores on study outcomes and confidence intervals. We will explore the distribution of the outcomes to investigate assumptions of normality in order to plan the analysis and sample size for the definitive trial. We will also analyze the cost of the intervention using data from the proformas completed by those delivering PSP. We will also estimate costs of TAU that participants receive using data from the Adult Service Use Schedule. These data will allow us to identify the main cost components which would need to be captured in a subsequent phase, full-scale trial.

Contemporaneous notes will be made during each interview and uploaded to NVivo analytical software before thematic analysis [45]. To generate an initial coding framework, an independent thematic analysis will be conducted on a sample of transcripts and a session held to discuss and refine the thematic structure. This initial coding frame may be further refined as the analysis progresses in response to emergent themes. Supported by NVivo, the coding framework will be applied to the data (indexing) with the aim of allocating all data to a theme. Where new codes are identified, previously coded transcripts will be re-checked to ensure that extracts of data are coded consistently throughout. Full copies of notes will be retained to ensure context is maintained. The aim of the analysis will be to describe the range of experiences and responses to the intervention, highlighting any patterns and divergences in respondent accounts which support the key research questions relating to feasibility, acceptability, and optimization of both the intervention and trial design.

### Progression criteria

Our criteria for determining the success of the feasibility study are: recruitment of at least 48 participants (80% of the target study sample of 60 participants); uptake of the low intensity intervention by at least 60% of participants in the active arm of the trial; and completion of follow-up interviews at six months by 75% of study participants. To determine the feasibility of calculating a cost analysis of health economics, we will record completion rates for the cost data and analyze them to determine what the main cost-drivers are likely to be in a full clinical trial.

### Ethics

Approval for the research has been given by the South Central Research Ethics Committee (Ref: 16/SW/0255) and from the Research and Development departments of the participating NHS Trust. We will ensure that the trial is conducted in full conformity with the current revision of the Declaration of Helsinki (last amended 13/09/2017. Version 4.0) and with the Medicines for Human Use (Clinical Trials) Regulation 2004 transposed into law from the EU Clinical Trials Directive 2001/20/EC, the EU Good Clinical Practice Directive 2005, and all current and future acts and requirements pertaining to its conduct.

Each study participant will be assigned a unique trial identification number at the start of the assessment process. This number will be written on all clinical assessment forms/datasheets and databases used to record data on study participants. A hard copy of a record sheet linking patient identity, contact details, and trial identification number for all participants will be kept at each site. It will be placed in the Investigator Site File, in a locked filing cabinet, separate from the paper Case Report Forms and other documents relating to a participant, which will be anonymized. Recorded data will be entered onto an electronic data management system that will use the trial identification number rather than the participant’s name or other information that could identify them.

## Discussion

This trial aims to generate data to test the feasibility of using a randomized trial to examine the effects of a low intensity intervention for people with personality disorders. Current guidance from the National Institute for Health and Care Excellence caution against the use of brief interventions for people with borderline personality disorder [4]. Despite this guidance, mental health services are under increasing pressure to try to ensure that more people with personality disorders receive evidence-based interventions and treatments. PSP is a new approach aimed at helping people with personality disorders, which is delivered to individuals rather than to groups of people, and is based on principles derived from longer-term interventions for people with personality disorders and recommendations of an expert panel of service users and providers.

PSP is a person-centered intervention which allows therapists and patients to develop an agreed plan for providing short-term psychoeducation and support that focuses on what matters most to the patient. Rather than attempting to treat the person’s personality, PSP aims to provide people with information about the nature of personality-related problems and psychological approaches derived from longer-term psychological treatments that may help people improve their mental health.

This trial has a number of strengths and limitations. It is designed to be pragmatic with broad inclusion criteria, limited exclusion criteria, and relatively short outcome assessment.

By enabling people with substance misuse problems, including substance dependence, to take part in the study we will be testing an intervention for an important group of people with personality disorders who are often excluded from existing interventions for people with this condition [46]. Unlike most trials of interventions for people with personality disorders, we will not make entry into the study dependent on a potential participant meeting validated criteria for the condition. Instead potential participants will need to meet criteria for “probable” personality disorders using the SASPD. We have decided not to include a more formal examination of personality disorders, because validated semi-structured instruments designed to make these assessments are lengthy (often taking > 1 h to complete). Furthermore, in a previous trial of problem-solving therapy for people with personality disorders in a secondary care setting, we found that nearly all those referred to the trial (650/682, 95.3%) met the criteria for personality disorders; those that did not still had significant personality pathology [47].

The trial combines the collection of both qualitative and quantitative data in order to make a comprehensive assessment of the acceptability and feasibility of the study design and in an effort to generate data that could help inform the design of a larger future explanatory trial.

We will be assessing the feasibility and acceptability of a range of outcomes. These are based on recommendations of both users and providers of services for people with personality disorders [48]. By including the patient version of the CGI Scale in the follow-up interview we will be able to examine whether the two treatment conditions are associated with negative as well as positive changes for participants. This information, together with the qualitative data we will be collecting, will enable us to examine concerns about possible negative effects of short-term interventions for people with personality disorders [4, 47].

Limitations of the trial include the absence of detailed information about the “type” of personality disorders which patients experience and the relatively short follow-up period. Our use of the SASPD will ensure that we will have reliable information about the severity of the personality disorders of study participants but will not enable us to describe the proportion of people who meet criteria for borderline, antisocial, and other specific personality disorders [32]. While this will limit our ability to compare the results of the trial with results of previous trials of borderline personality disorder, we have purposefully adopted an approach to the diagnosis of personality disorders that will form the basis of revised World Health Organization criteria which are primarily based on severity of interpersonal functioning and sense of self rather than type of personality-related problems [49].

We decided to restrict the length of follow-up in the study to six months because our primary aim was to establish the rate of recruitment in the trial as well as the acceptability and uptake of the intervention we are testing. However, personality disorders are long-term conditions and a future larger-scale explanatory trial would benefit from having a longer-term follow-up of 12–18 months.

This trial faces a number of challenges which will need to be overcome if we are to recruit to target. In addition to pressures of workload and other factors that may deter clinicians from referring people to clinical trials, staff may be concerned about the impact of their patient being randomized to “treatment as usual,” which many consider unsatisfactory. Clinical staff may prefer the option of referring patients for assessment for psychological treatment from a psychologist or psychotherapist. However, such services typically have long waiting lists and referral criteria which exclude some of the patients we aim to recruit to this study (such as those judged at risk of severe self-harm and those with coexisting substance misuse). Potential participants may also be unhappy about the prospect of being randomly allocated to the two arms of the trial and may have a preference either for continuing to receive care from their existing team or for receiving the new intervention that we are testing. For this reason, we have designed patient information sheets to reflect the similarities as well as the differences between the two treatment conditions in the trial. For instance, both treatment conditions aim to work with people in the short to medium term with a view to discharging the patient from secondary care mental health services. Participants in both arms of the trial will also have access to other secondary care services including crisis helplines, review of medication, and referral to longer-term psychological therapies if these are indicated. We will be examining the views of study participants on this and other aspects of the trial design as part of the process evaluation we are conducting in parallel to the randomized trial.

## Conclusions

This trial has the potential to increase the quality of care that people with personality disorders currently receive by paving the way for an explanatory trial of a low intensity treatment that would enable more people with personality disorders to access and potentially benefit from effective support aimed at helping them achieve better mental health and social functioning.

### Trial status

Recruitment is ongoing (50 participants recruited as of end of May 2018).

## Abbreviations

CGI: Clinical Global Impression Scale
CONSORT: Consolidated Standards of Reporting Trials
DSM: Diagnostic and Statistical Manual of Mental Disorders
EQ-5D-5 L: EuroQol Group 5-Dimensions 5-level
EU: European Union
IPDE: International Personality Disorder Examination
PSP: Psychological Support for Personality
SASPD: Standardized Assessment of Severity of Personality Disorder
TAU: Treatment as usual
